# Glutathion-S-Transferases (GSTs) and Parkinson’s disease in a MPTP-induced C57BL/6 mouse model

**DOI:** 10.1186/1750-1326-8-S1-P30

**Published:** 2013-09-13

**Authors:** Elcin Deniz Ozdamar, Benay Can-Eke

**Affiliations:** 1 Ankara University Faculty of Pharmacy Department of Toxicology, Ankara, Turkey

## 

Parkinson’s disease (PD) is the second most common neurodegenerative disorder after Alzheimer disease and affects up to 2% of individuals after the age 65. It is characterized by progressive motor decline and loss of dopaminergic neurons from the substantia nigra (SN) leading resting tremor, rigidity, bradykinesia and postural instability. Differential characteristics of PD pathology is the loss of dopaminergic neurons and the presence of intracellular protein aggregates. The etiology of the disease is still unknown and it is likely due to a multifactorial interaction of genes and the environmental factors on the background of ageing.

The impact of enzyme activities, gene expressions and genetic polymorphisms in GSTs on PD has received particular interest since these enzymes play an important role in the detoxification and metabolism of several environmental chemicals and xenobiotics which may be associated to PD. Patients with idiopathic PD appear to have reduced capacity for detoxification and/or metabolism of certain environmental compounds such as cigarette smoke and pesticides.

Therefore we aimed to examine GST theta, mu, pi and total GST activities by the use of ENPP, EAA, DCNB and CDNB as substrates, respectively in a MPTP induced animal (mouse) model of Parkinson’s disease. In the same animal model we also investigated the gene expression levels of GSTM1 and GSTP1 using the method of qPCR.

As a result, we observed an increase in cytosolic GST teta (GSTT) and cytosolic total GST activity which are statistically significant. qPCR results indicated an significant increase for GSTP1 mRNA expression level.

